# Study protocol for a pragmatic parallel-group randomised controlled trial to evaluate the effectiveness of coaching with an online intervention, compared with the online intervention alone, for families of children who have experienced developmental regression

**DOI:** 10.1136/bmjopen-2025-105615

**Published:** 2026-06-10

**Authors:** Wan Hua Sim, Monica Leo, Helen M Bourke-Taylor, Charmaine Bernie, Amanda Brignell, Alexandra Ure, Kirsten Furley, Adele Maree Fahey, Anoo Bhopti, Sorcha Odgers, Leah Picking, Jue Xie, Ling Wu, Marie B H Yap, Katrina Williams

**Affiliations:** 1 Department of Paediatrics, School of Clinical Sciences, Monash University, Clayton, Victoria, Australia; 2 School of Psychological Sciences, Turner Institute for Brain and Mental Health, Monash University, Clayton, Victoria, Australia; 3 Departmental Paediatrics, Monash Children’s Hospital, Clayton, Victoria, Australia; 4 Department of Occupational Therapy, School of Primary and Allied Health Care, Monash University, Frankston, Victoria, Australia; 5 Southern Cross University, Bilinga, Queensland, Australia; 6 Psychology & Specialist Services, Monash Health, Clayton, Victoria, Australia; 7 Independent Researcher, Brisbane, Queensland, Australia; 8 Action Lab, Department of Human-Centred Computing, Monash University, Clayton, Victoria, Australia

**Keywords:** Parents, Psychosocial Intervention, Developmental neurology & neurodisability, Rare Diseases, Telemedicine, eHealth

## Abstract

**Introduction:**

Developmental regression is when children lose one or more skills they have established. Families caring for these children need timely recognition to assist diagnosis and tailored interventions. Families also need support to develop practical skills for caregiving and strategies to promote family well-being and community participation. Given the high caring demands, flexibly delivered approaches are needed to accommodate family routines. Online delivery of health-related interventions that provide coaching, information, or both has been found to be a feasible and effective option for families. Family Focus is a new family-centred online programme, co-designed with parents and family advocates, clinicians, and researchers to support and empower primary carers.

**Methods and analysis:**

This study is a prospective, pragmatic randomised controlled trial comparing the effectiveness of online parent coaching plus Family Focus (Coaching+FF) to Family Focus alone (FF) for primary carers of children experiencing developmental regression. A sample of 56 families will be randomised in a 1:1 ratio. Outcomes are assessed at baseline, post-intervention and 12-month post-randomisation. The primary outcome is parental stress symptoms at post-intervention. Secondary outcomes include parental depressive and anxiety symptoms, parental engagement in health-promoting activities, family empowerment, family quality of life and child global health outcomes. The study will also examine the uptake and acceptability of specific coaching and FF components and explore the facilitators and barriers to their delivery and implementation.

**Ethics and dissemination:**

Ethics approvals were obtained from the participating organisations (Monash Health HREC/107806). Informed consent is obtained from parents/guardians of children prior to study enrolment. Study findings will be disseminated through peer-reviewed publications, conference presentations and lived experience agencies.

**Trial registration number:**

ISRCTN25513446.

**STRENGTHS AND LIMITATIONS OF THIS STUDY**

- This will be the first study using a randomised controlled trial and mixed-methods design to assess the effectiveness of an online programme for reducing parental stress, with and without coaching, in parents of children with developmental regression.
- A key strength of the study is the codesign of the programme with an advocacy and research-driven non-profit organisation and families of children experiencing developmental regression, in partnership with clinician researchers.
- The time interval between onset of developmental regression and the start of the intervention will vary among participants. As participants enrol one at a time in this trial, it is impractical to use stratified randomisation based on different time intervals.
- While the implementation of strategies to recruit, engage and retain participants has been included, lower than expected recruitment would limit the scope of the analyses and conclusions.

## Introduction

 Developmental regression (hereon, ‘regression’) is broadly described as the loss of previously established abilities or skills in children. Early signs of regression may not be apparent to parents and thus may result in delayed presentations to child health services.^1^ In particular, very young children, particularly those with delayed skill acquisition, have fewer abilities to lose, and so their regression may be less obvious.^2^ Families may also encounter delayed assessment, as there is often no specific pathway established for addressing presentations of regression unless the regression is sudden and severe, indicating a potential neurological emergency. This can lead to delayed care, increased stress for families and missed opportunities for early intervention.

Children presenting with regression may have a neurodevelopmental condition^3^ or a childhood dementia condition—an umbrella term that refers to a group of progressive neurodegenerative diseases.^4^ Both types of conditions will benefit from early identification and prompt intervention. Timely investigation may identify a diagnosis and empower a family with knowledge to make informed decisions about interventions and future reproductive choices, as well as offer insights for extended family members. Compared with observing delayed development from the outset, witnessing a child lose skills they had attained as the onset of a condition has been linked with increased levels of parental distress, particularly given the uncertainty around their child’s diagnosis and prognosis following skill loss.^5^ When a treatable cause is not found, the distress and shock families experience can be heightened.^6^ Families caring for children experiencing regression require timely recognition and support, along with the skills to promote their child’s care and development. They also need support to navigate services, maintain family health and well-being, and foster community inclusion and participation.^7^ Health education, psychoeducation and health promotion programmes are essential in supporting parent mental health^5 8^ and play a fundamental role in early intervention and the overall support provided to families of children experiencing regression.^6^

Family-centred approaches that build the capacity of parents to care for their child are considered best practice when working with children and families.^9 10^ One capacity-building approach commonly used within the context of paediatric practice is coaching with parents. Research has shown coaching can be an effective method for supporting progress towards parent goals and improving parental sense of competence, confidence and engagement.^11 12^ A recent review of coaching provided by allied health professionals in paediatrics highlighted inconsistency in definitions of coaching.^8 13^ Akhbari Zeigler and Hadders-Algra^13^ recommend that parent coaching should refer to support for families that is relationship-based, family-centred and where the overarching goal is the optimal participation of the child and family in daily life and empowerment of the family to support the child’s development. During coaching it is recommended that a collaborative relationship be developed, in which parents are supported to make informed decisions through guidance from the coach.^13 14^ In this context, coaching incorporates joint planning, observation, action, reflection and reciprocal feedback.

However, families with intensive caregiving tasks can experience barriers to accessing in-person supports such as coaching, health or psychoeducation programmes or specific skill training to support their child’s development.^15 16^ Technology-assisted parenting programmes have been recently recognised by the United Nations as a way of delivering support for families, made possible by increased parental digital literacy and accessibility.^17^ Consumer surveys of Australians in 2021 and 2022 revealed a significant increase in consumers who are not only open to but are actively seeking healthcare through videoconferencing, remote monitoring and asynchronous communication with clinicians.^18^ This rise in consumer demand for virtual care indicates a growing acceptance and comfort with virtual healthcare.^18^ Flexible access to technology-based services is an opportunity to increase timely access to intervention^19^ to improve health outcomes for children experiencing regression and their families.

Evidence is rapidly emerging that online interventions are effective in improving parenting, supporting parent health, and promoting parent mental health and wellbeing.^20^,^22^ Research also shows that interventions led by parents can improve the physical and mental health of children.^23^ A recent systematic review and meta-analysis of interventions for parents reported that online interventions that are combined with health professional contact or consultations (blended care) were adopted in 22 of the 30 studies in the review.^20^

Blended care is fast becoming common practice in the current technological age, combining online components for assessment, psychoeducation, skills training and therapist guidance provided either in person or virtually.^24^ This approach enhances the support provided for applying skills. A systematic review reported that guided digital mental health interventions for youth and young adults, using a combination of asynchronous (online content) with synchronous (in-person online) support, had higher rates of completion.^25^ While past studies did not explicitly report effect sizes within guided groups (eg, completers vs non-completers), the literature suggests that completers in guided interventions likely achieve better outcomes due to higher adherence to the intervention content and procedure.^26^ A recent three-arm randomised controlled trial of a web-assisted intervention for parents of children with attention-deficit/hyperactivity disorder and oppositional defiant disorder found that the arm that received a guided intervention was more effective in reducing blinded clinician-rated externalising symptoms than the unguided or usual care control groups.^27^ However, the study noted that other similar studies of guided online interventions had contradictory findings and that further research is needed to compare guided and non-guided online interventions.

To empower and support primary carers of children experiencing regression, we designed an online, self-paced programme—Family Focus (FF). The programme’s content and delivery were informed by insights gained from a review of the literature of parent-focused or family-focused online, self-paced support to identify the most important information for parents caring for children with complex developmental needs.^20 28^ This novel, codesigned online multimedia programme is designed to build on parents’ strengths, foster resilience, enhance coping strategies and well-being and promote meaningful participation for both the child and family in daily life and the community. When combined with one-on-one parent coaching support (Coaching+FF), families might engage with a personalised family-centred experience. To support this, we drew on existing literature and models on coaching^29 30^ and developed a protocol for offering individualised, online coaching to parents.

### Aim and objectives

This study aims to evaluate the effectiveness and uptake of a blended approach (Coaching+FF) for primary carers (hereon, ‘parents’) of children experiencing developmental regression, compared with the online programme alone (FF). By using an active comparator in this study, we aim to generate clinically relevant and realistic estimates of the effects of blending coaching and the online intervention,^31^ over and above the effects of the online intervention alone. This approach acknowledges the urgent need for support among families managing child regression concerns and provides a practical framework for evaluating new intervention strategies in this context.

In comparison to parents who engage with FF without coaching (‘comparison group’), the objectives are to determine:

1. The effects of Coaching+FF (‘intervention group’) on the primary outcome of parental stress symptoms.
2. The effects of Coaching+FF (‘intervention group’) on the secondary outcomes of (1) parental anxiety and depressive symptoms, (2) family empowerment, (3) parental engagement in health-promoting activities, (4) family quality of life and (5) child’s global health outcomes.

We will also explore the proportion of parents in the FF group who transition from above to below the clinical thresholds for stress, anxiety and depressive symptoms at post-intervention. In addition, the uptake and acceptability of online parent coaching and the FF programme will be examined by assessing recruitment and retention rates, user engagement metrics, coaching uptake, programme usage data and participant feedback on perceived usefulness and satisfaction at post-intervention.

We hypothesise that, compared with parents in the FF group, parents in the Coaching+FF group will report lower levels of parental stress, anxiety and depressive symptoms, and greater improvements in family empowerment, engagement in health-promoting activities, family quality of life and child’s global health outcomes. These differences are expected to emerge at post-intervention and follow-up. Further, we anticipate that parents would find online coaching and the FF programme satisfactory and acceptable.

## Methods

### Study design

The study is a parallel, two-armed, superiority randomised controlled trial (RCT) that examined the effectiveness and uptake of online parent coaching combined with FF. The study is registered with the International Standard Randomised Controlled Trial Number registry (ISRCTN25513446). Data will be collected at baseline (pre-randomisation, T0), post-intervention (6-month post-randomisation, T1) and follow-up (12-month post-randomisation, T2).

### Study setting

The RCT will be conducted with families of children referred for assessment of loss of skills to the Developmental Regression Clinic at Monash Children’s Hospital in the Australian state of Victoria. The clinic is a public specialist tertiary service jointly funded by the hospital and a Medical Research Future Fund (MRFF) grant from the Australian government. Referrals are accepted from GPs or other medical specialists in Victoria. Eligibility for the clinic is based on suspected developmental regression, defined as parent- or clinician-reported loss of previously acquired skills in at least one developmental domain (communication, motor, social engagement or daily living) within the preceding 18 months. This is a clinical presentation rather than a discrete entity with a single diagnostic code, and it serves as the trigger for a structured multidisciplinary assessment to clarify the underlying aetiology. Regression is operationalised through detailed developmental history, supported where possible by documented prior functioning (eg, reports, home videos or clinician observations).

Children referred to the clinic are seen by a multidisciplinary team consisting of a paediatrician, an occupational therapist clinic coordinator, a neuropsychologist and/or a speech pathologist. Each child undergoes a comprehensive assessment consisting of parent-report, direct observation and standardised measures, with the specific assessment battery tailored to each child’s age, abilities and presenting features. The multidisciplinary team conducts weekly case conferences with neurology and genetic specialists to plan first-line investigations and early management. Children are reviewed at approximately 8 weeks, 6 months and 12 months. At each review, investigation results and clinical progression are reviewed, and further targeted investigations are recommended where an explanatory diagnosis remains unclear. Possible explanatory diagnoses considered include autism, neurodegenerative conditions (eg, Batten disease), developmental epileptic encephalopathies, recognised genetic conditions (eg, Rett syndrome), and other rare or emerging genomic or mitochondrial conditions, including possible phenotypic expansions. Where autistic features emerge over the follow-up period, an autism-focused assessment may be undertaken alongside standard developmental assessments and medical investigations. The clinic explicitly seeks to minimise diagnostic overshadowing by continuing to investigate other potential causes of regression even when autistic features are present.

### Eligibility criteria

Parents of children aged between 1 and 15 years with loss of skills in one or more developmental domains (communication, motor, social engagement or daily living) within the 18 months prior to their first visit to the Developmental Regression clinic are the target population. Other eligibility criteria include residency in Victoria, Australia, comfort in reading English at Grade 7 level, access to an internet-enabled device and an active email account.

Participants are not eligible if: (1) the child’s history of loss of skills cannot be corroborated through developmental surveillance information, clinical assessment or review of video/audio recordings, or (2) the regression is judged to have occurred primarily in the context of significant environmental adversity such as documented abuse, neglect or severe household adversity or (3) the child’s condition warrants prioritisation of medical intervention and stabilisation over research participation.

### Recruitment

Eligible parents of children who are currently attending, or have attended, the Developmental Regression clinic will be invited to participate in the study. The consent procedure includes the clinic/trial coordinator sharing and explaining to the parent using the appropriate information and consent forms (see online supplemental material for an example). The exact timing of the approach for research consent will be determined by consideration of the parent’s physical, cognitive and psychological capacity at the time, as well as the practicality of the circumstances, to ensure that obtaining informed consent is not burdened by high impact on time and other resources that could compromise care. Prospective participants will also be presented optional consent options, including those consenting their data to be recorded in a skill loss registry or shared with other registries. Participant enrolment will start in January 2026, and the last participant is expected to be enrolled in December 2026.

Participants can also nominate another carer of their child or a support person to access the FF programme. The additional carer or support person will be invited to give their own consent to create a login for the programme but will not be required to complete assessments that are expected of participants.

### Interventions

#### Family Focus

The FF programme was developed specifically for parents caring for children experiencing developmental regression. It is organised into a foundational knowledge and skill-building package consisting of 10 self-paced online modules, integrating a blend of text, images, interactive and auditory content. Six of these modules’ topics are also featured in podcast content.

All parent participants will receive their own login to access FF for the duration of the trial. To enhance programme engagement, nudges and reminders in the form of emails and text messages will be delivered based on parents’ preferences.

Importantly, the FF programme was constructively aligned with specific target behaviours that were expected to change and operationalised as parent outcomes. The programme was developed through a five-step co-design process. First, a review of the literature on family resilience and participation, parent stress, parent empowerment, and child outcomes identified parental stress and mental health as critical intervention targets given their high prevalence and demonstrated impact on family functioning, particularly in families of children with developmental disabilities.^2028 32^,^35^ Second, thematic mapping of the literature, lived experience inputs, and best-practice guidelines^36^,^39^ identified key protective and risk factors influencing family resilience and participation, resulting in five module domains: (1) parent mental health and support, (2) parent skill and family culture, (3) child development and participation, (4) community participation and (5) siblings and family. Third, module content, learning objectives and skill targets were systematically aligned with intended outcomes—including reductions in parental stress—through iterative forward and backward mapping. As part of the codesign consultation phase, parents with lived experience were also consulted about whether access to a coach with allied health training, in addition to an online module package, would provide added benefit and how such support could be delivered. Fourth, content was co-developed and reviewed by authors who are allied health professionals and by parents with lived experience, to ensure relevance and appropriateness. In the prototyping stage, parents with lived experience provided feedback on the layout and functionality of specific elements of the FF programme (eg, individual modules and dashboard functions) and the proposed coaching options (eg, focus, frequency and supporting features in the dashboard). Finally, the programme was delivered as an online package with a coaching component, informed by iterative feedback during the development process. See table 1 for an overview of the module domains and content.

**Table 1:** Module domain, module title and brief outline of Family Focus online content

Domain	Title	Outline
1. Parent mental health and support	Module 1: Keeping yourself strong and healthy	Introduces the concepts of well-being and resilience, and explores what parents might experience when caring for a child with complex developmental needs
	Module 2: Being healthy and bringing support towards you and your family	Offers practical strategies to support parental mental health and well-being, including managing routines, bringing supports towards the family and engaging in health-promoting activities
2. Parent skill and family culture	Module 3: Building parenting skills to promote a healthy family culture	Explores ways that parents can help everyone in the family feel safe, secure and healthy, such as through family routines
	Module 4: The role of parents and ways to manage now and in the future	Considers the roles and responsibilities of a parent caring for a child with complex developmental needs, and presents practical resources for managing different challenges the family may face
3. Child development and participation	Module 5: Supporting your child’s participation at home	Introduces the meaning of participation and offers strategies to support a child’s participation in preferred activities
	Module 6: Supporting your child’s communication and social interaction	Supports parental reflection on their child’s needs and shares ideas for supporting their child’s communication and socialising at home
	Module 7: Supporting your child’s development in play, self-care and movement	Presents strategies to support a child’s development in the areas of play, self-care and movement including sensorimotor development
4. Community participation	Module 8: Being a part of the community and enjoying family life	Looks at common barriers families face in getting out and about, and explores ways families can stay connected with their communities
	Module 9: Making positive connections with old and new communities	Talks about the challenges families may face in participating in cultural and community activities and the strategies families can use to navigate these challenges
5. Siblings and family	Module 10: Supporting siblings and nurturing connections	Considers the needs of siblings of children with complex developmental needs, the impact and opportunities these challenges present, and shares ideas for offering support

**Figure 1:**
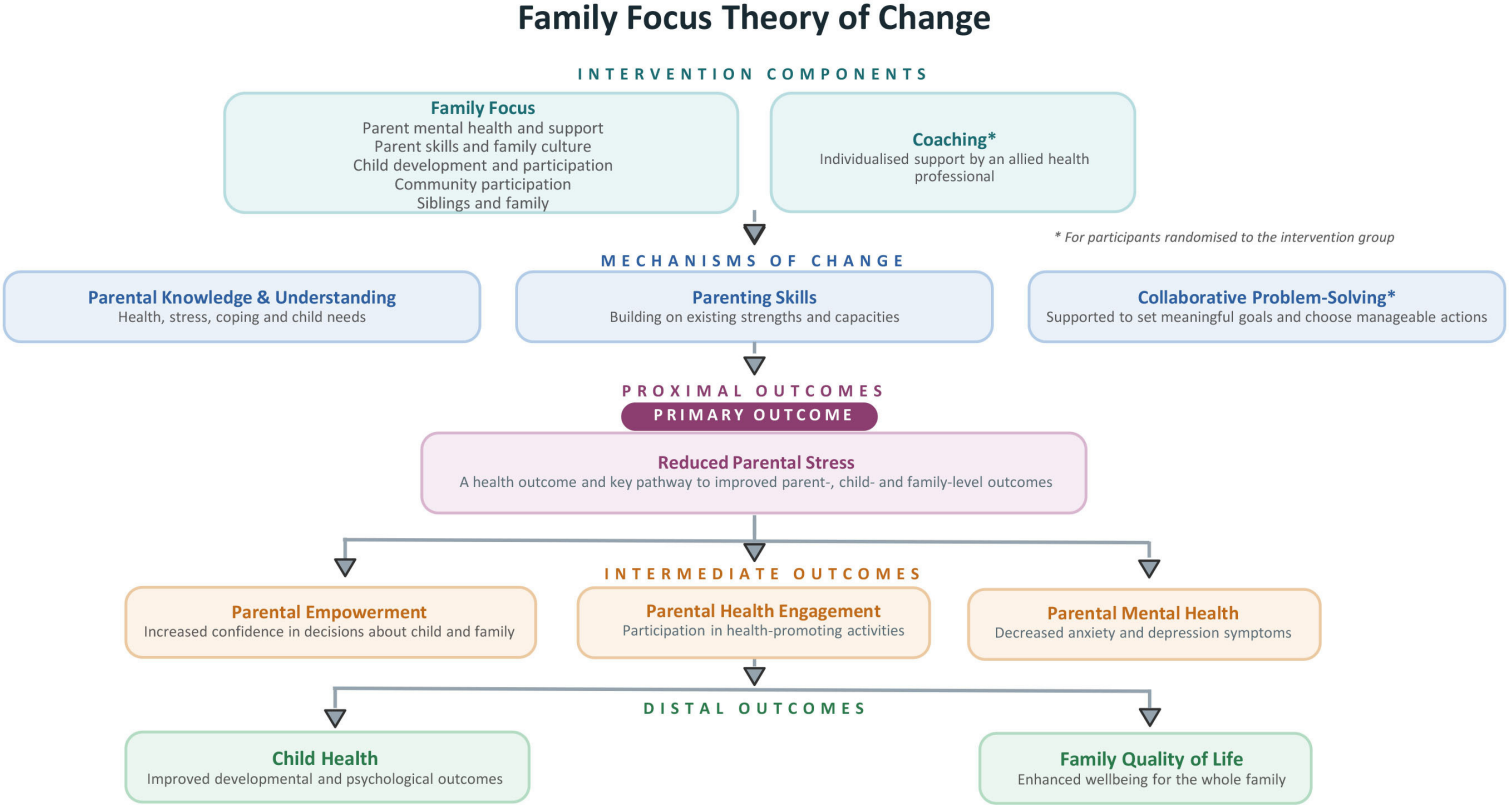
Theory of change.

The FF intervention provides educational resources and practical strategies targeting parent mental health, healthy behaviours (ie, sleep and physical activity) and the health, well-being and participation needs of all family members. Content focuses on building skills for managing caregiving responsibilities, establishing sustainable healthy home routines and supporting engagement in family and community activities. These components are designed to enhance parent empowerment and competencies, increase parental knowledge, skills and understanding to support adjustment to their child’s needs and caregiving requirements, while promoting the well-being of the whole family. Through these behaviour change targets, the programme aims to reduce parental stress as its primary, proximal outcome, recognising that lower stress is both a meaningful health outcome and a key pathway to improved child and family outcomes (see figure 1). As parents access support, resources and opportunities to build on their existing strengths, parents shape conditions that promote responsive caregiving, enhance their empowerment and knowledge around their child’s specific needs and decisions affecting their child and family, and support active engagement in activities that promote their own health and well-being, thereby reducing parental anxiety and depression (intermediate outcomes). Over time, these changes are expected to contribute to better child physical and mental health and an improved quality of life for the whole family (distal outcomes).

In the intervention arm, FF is combined with coaching to augment these effects. Coaching offers guided goal setting, individualised problem solving and accountability over time. By providing tailored support and opportunities to apply and refine skills in parents’ real contexts, coaching is expected to strengthen key mechanisms of change.^13 14 29^ Consequently, FF plus coaching is hypothesised to produce greater and more sustained reductions in parental stress, larger gains in parent empowerment and well-being (intermediate outcomes) and, over time, more pronounced improvements in child participation, functioning and overall family quality of life (distal outcomes) compared with FF alone.

#### Coaching

Coaches will be allied health professionals with previous experience in coaching families of children with developmental differences in healthcare or disability settings. They will receive training on the FF programme content to ensure familiarity with the materials for parents. In addition, they will be trained on procedures such as booking of sessions, scope of the coach role and elements of sessions to be documented (eg, date, duration, parent goals and identified actions), as well as the principles underpinning the coaching model in this trial, which include:

- Relationship based, collaborative and family centred.
- Goal generating, in line with the family’s needs, priorities, preferences and resources.
- Goals focused on overall child and family functioning, well-being or participation.
- Aiming to optimise empowerment and participation of the family as they provide support for their child with developmental regression and other family members.
- Coaching conducted in a strengths-based approach with joint planning, observation, reflection, action and feedback.

The role of the coach is to create opportunities for the parents to establish goals important to them and their family, reflect on what information and resources that would assist progress towards the goal, and to encourage parents to identify actions they can take. When parents identify goals that are related to content within the online programme, the coach will support reflection and discussion of the relevant online content and guide the parent to determine ways in which potential strategies can be implemented into family routines. Coaches will not provide direct therapy for the child and will recommend families consult with their community-based therapists for child-related therapy questions.

A fidelity checklist for the coaching approach will be developed and will include the expected coaching behaviours that reflect the key principles named above. Where the participant provides consent to audio-recording or video-recording, the coach will record the session to self-evaluate performance on the fidelity checklist post session. These recordings will be randomly sampled and rated by a research team member with experience in this coaching approach to ensure fidelity to the protocol and consistency across coaches in approach.

### Patient and public involvement

The FF programme was designed by bringing together perspectives and needs of parents and the expertise of researchers and clinicians from occupational therapy, speech pathology, psychology, developmental education, paediatric medicine and human-centred computing. Furthermore, family advocates from the Childhood Dementia Initiative are project partners, which ensures that the programme is relevant and acceptable to families of children with some of the most profound or complex forms of regression. Across a series of interviews and iterative workshops between 2023 and 2025, 14 families with lived and living experience played a significant role in identifying the target users’ needs, suggesting content topics, formats and features to include in the online programme and providing feedback on prototypes. Three additional parents of children with disabilities recruited from the community provided further insights and contributed to the development of podcast content that provide bite-sized, audio episodes on key topics. The author team of the FF programme content and this manuscript included a parent (AMF) with lived experience of caring for a child with developmental regression. Combining and triangulating systematic review findings^20 28^ with direct insights from families with lived experience has helped us create a programme that would resonate with and benefit its users.

The families who were involved in the codesign process will not be recruited to the current study. Details regarding the codesign process and findings will be communicated in a separate publication.

### Trial arms

#### FF arm

Parents in the comparison group will access the FF programme through an online portal but will not be able to request one-on-one coaching sessions. Instead, they will receive up to 12 prompts over 6 months (through SMS/email). The prompts will invite parents to think about the type of information or support that would be helpful to them and also suggest specific module content or other resources on the portal. These prompts will serve as a retention strategy and will support parents to navigate resources available in the online programme in the absence of a coach. Parents in this group will complete the same baseline, post-intervention and follow-up assessment as the intervention group.

#### Coaching+FF arm

Parents in the intervention group will access up to twelve 45-minute one-on-one coaching sessions via videoconference over a 6-month period, in addition to access to the FF programme. Parents will be offered flexibility with scheduling coaching sessions, which can occur weekly or at wider intervals to suit their needs and preferences. As the role of the coach in these sessions is to support the parent in identifying what they want to work on or change (the goal), parents are not required to have viewed a particular module or podcast in the programme before requesting a coaching session. After each coaching session, the parent is invited to complete a 4-item questionnaire to provide feedback on what is working well and identify areas for improvement in the sessions with their coach.

Participants and their children in both study arms may continue to receive any standard or necessary medical care as determined by their healthcare providers throughout the study period. Participants can discontinue their participation in the study at any point.

### Study procedures

#### Enrolment and randomisation

Eligible parents of children assessed through the research-embedded Developmental Regression clinic will be provided with information about the intervention trial and invited to participate. For those who consent to this study, if data on the outcome measures were collected as part of their first assessment or a follow-up review at the clinic within the past 6 months (whichever is closer to their study enrolment), the data will be transferred and used as their baseline data (T0) in the trial. Specifically, direct identifiers (eg, names) will be replaced by a code before participant data are transferred to a research-only database using REDCap, a secure, web application to support data capture.^40^ A 1:1 randomisation allocation sequence will be computer-generated within REDCap to allocate parent participants to the FF group or Coaching+FF group, thereby ensuring that participants, coaches, trial coordinator, outcome assessors and the research team analysing the data are concealed from the group assignment until baseline assessment and allocation are completed. Outcome assessors (clinicians completing assessments of the child) will remain blinded to group allocation across the assessment timepoints.

**Figure 2:**
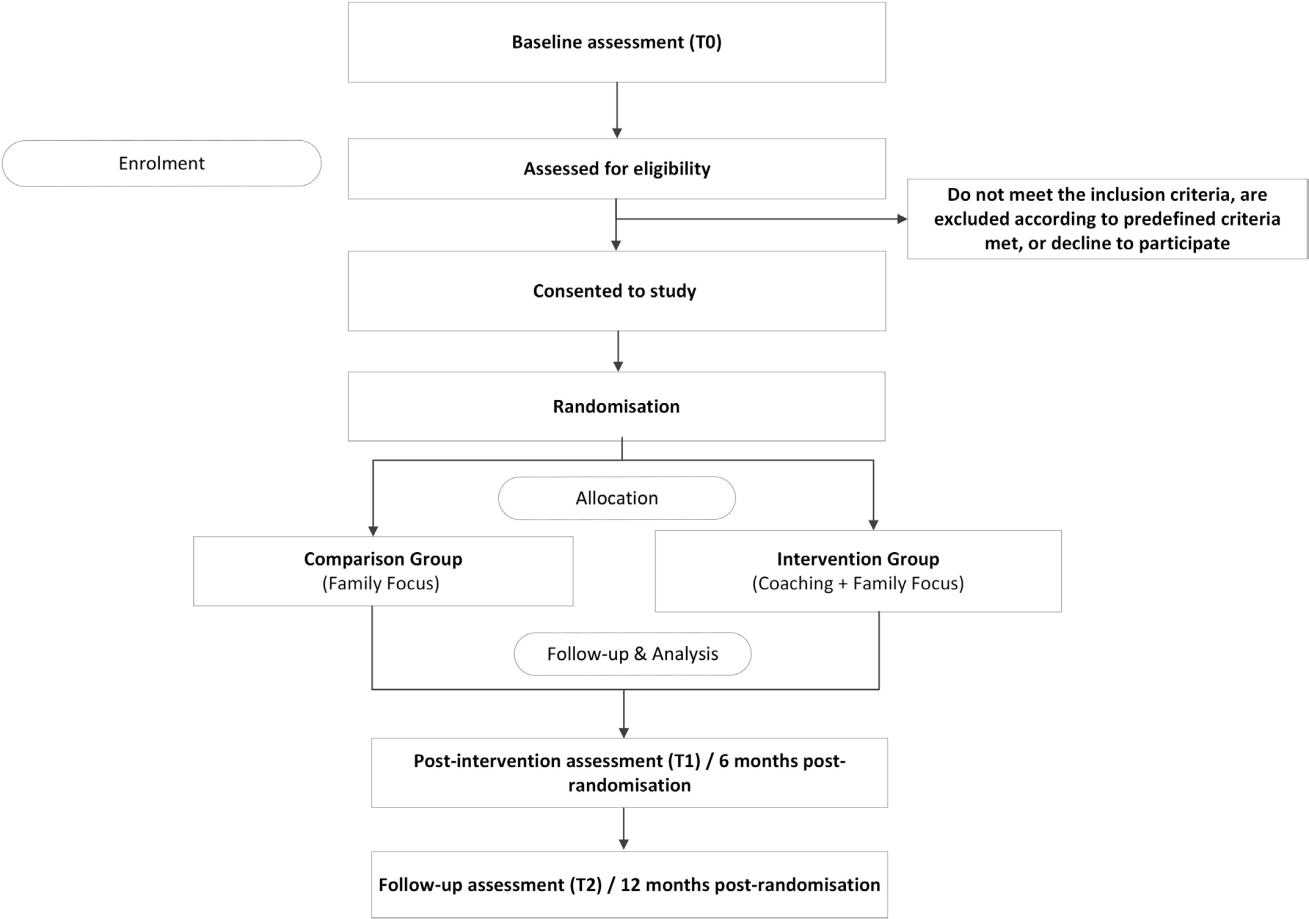
Participant flow diagram.

Participants will also complete post-intervention (T1) and follow-up (T2) assessments via online questionnaires hosted in REDCap. Figure 2 outlines the participant flow.

#### Blinding

To minimise bias and possible contamination, the coaches in this trial will be employed by Monash University and will be independent of the Developmental Regression clinic. The coaches working with the participants in the Coaching+FF group will not be in contact with participants in the FF group.

The FF programme will be implemented through the Induk (https://induk.io/) technical infrastructure, which securely encodes participant accounts using anonymous unique identifiers for each participant. This mediated approach ensures that potential bias from administrators or the research team is effectively eliminated. Given that this is a pragmatic trial, it is not possible or appropriate to blind parent participants (ie, parents would know if they would access coaching).

The questionnaires for collecting primary outcome data and other parent-reported outcomes are administered and captured by REDCap, thereby maintaining the blinding of researchers to treatment allocation and ensuring consistent data collection.

#### Sample size

This study will enrol eligible families of children seen at Monash Children’s Developmental Regression Clinic who have consented to taking part in this intervention trial.

As there was a lack of available effect size estimates for interventions targeting families of children with regression, we used effect sizes from online interventions that aimed to improve mental health, stress and well-being of parents of children with a disability or chronic medical condition.^20^ To calculate the sample size required for *F-*tests (analysis of covariance (ANCOVA) and analysis of variance (ANOVA)), G*Power 3.1.9.7 software was used.^41^ To detect a large effect (*f*= 0.5) in the primary outcome (ie, parental stress) with a power of 80% and α of 0.05 (two-tailed), a sample size of 43 parents is required. To allow an attrition of approximately 30% between baseline (T0) and follow-up assessment (T2), a sample size of 56 parents (28 per group) is required.

### Outcomes and other measures

Outcome measures will be administered at baseline (T0), post-intervention (6 months post-randomisation, T1), and follow-up (12 months post-randomisation, T2).

The primary outcome of the study is parental stress. The self-report, seven-item Stress scale from the Depression Anxiety Stress Scales^42^-short form (DASS-21) will be used to assess nervous arousal, restlessness, impatience, irritability, overactivity and agitation. Each item is rated on a 4-point scale (0=never, 1=applied to me to some degree, or some of the time (sometimes), 2=applied to me to a considerable degree, or a good part of the time (often), or 3=applied to me very much, or most of the time (almost always)).

Secondary outcome measures include the following:

1. Parental depressive and anxiety symptoms, assessed by the Depression and Anxiety scales of the DASS-21,^42^ with higher scale scores indicating higher levels of depression and anxiety symptomatology, respectively.
2. Family empowerment, using the 34-item Family Empowerment Scale (FES).^43^ Each item is rated by the parent on a scale ranging from 0=not applicable to 5=very often. An overall score can be computed to measure parents’ attitudes, knowledge and behaviours regarding empowerment in relation to their child. Higher scores indicate higher levels of empowerment.
3. Family quality of life, measured by the 25-item Beach Center Family Quality of Life Scale (FQOL)^44^ to capture families’ perception of their quality of life across five domains: family quality of life: family interaction, parenting, emotional well-being, physical/material well-being and disability-related support. The scale uses a 5-point scale (1=very dissatisfied to 5=very satisfied), with higher scores indicating higher levels of family quality of life.
4. Parental engagement in activities that promote their health, obtained through the 8-item Health Promoting Activities Scale^45^ (HPAS). Parent respondents estimate the frequency of their participation along a 7-point scale where 1=never to 7=once or more every day, and scores are totalled for an overall score. Higher scores indicate more frequent participation in activities that promote health.
5. Child global health outcomes, using the Clinical Global Impression Scale.^46^ Originally developed for assessing patient outcomes in mental health conditions based on functional impact and clinical meaningfulness, the scale has been employed in studies in the context of various medical and neurodevelopmental conditions as a global rating of severity and change in interventional studies.^47 48^ In this trial, two of the three scale items will be used. Using a 7-point scale, the clinician will rate the child’s illness severity (CGI-S) and improvement or change (CGI-I) relative to their experience with other children with the same condition.

#### Other measures

To assess programme uptake and acceptability, recruitment and attrition rates, along with any available information on the reasons for choosing not to participate or to withdraw at each stage of the trial, will be tracked and documented.

Data associated with participant engagement with the FF programme will also be collected. These include pages visited (home/resource page, dashboard, modules, podcasts, focus board, etc), number of podcasts listened to, number of tipsheets viewed or number of downloads, activities completed within modules (percentage), and the number of goals or focus areas identified and worked toward. Acceptability of the FF programme will be assessed by self-report questionnaires on user experience (usability, usefulness and satisfaction ratings).

Quantitative and qualitative data on participant engagement with online coaching and coaching acceptability will be collected from participants in the Coaching+FF group. Engagement data includes the frequency of coaching sessions scheduled versus completed, and the percentage of coaching sessions elected by participants out of a maximum of 12. Acceptability of online coaching will be assessed by participants’ ratings on perceived ease of accessing the coach, satisfaction with the coaching support and the usefulness of the coaching sessions in enhancing their confidence to support their child and family.

Coaches’ fidelity to the coaching protocol for this study will also be examined through a random sampling of session recordings for rating by a research team member familiar with the coaching approach. Data will also be collected on time spent on coaching and associated tasks to assess feasibility. To better understand the facilitators and barriers to the delivery and implementation of online coaching and the acceptability of specific intervention elements in the context of developmental regression, separate one-on-one semi-structured interviews will be conducted with parents and coaches at post-intervention.

The schedule of enrolment, interventions and assessments is outlined in table 2.

**Table 2:** Schedule of enrolment, interventions and assessments

Timepoint	Enrolment	Allocation	Post-randomisation	
	T0 (baseline)		T1 (post-intervention)	T2 (6-month follow-up)
**Enrolment**				
Eligibility screen	X			
Informed consent	X			
Randomisation		X		
**Interventions**				
Family Focus		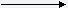		
Family Focus+Coaching		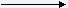		
**Assessments**				
***Outcomes***				
DASS-21	X		X	X
FES	X		X	X
FQOL	X		X	X
HPAS	X		X	X
CGI-S	X		X	X
CGI-I	X		X	X
***Other data***				
Skill regression information	X			
Antenatal and pregnancy information	X			
Developmental history	X			
Physical and medical examination or history	X			
Support and services information	X		X	X
FF engagement		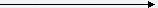		
FF acceptability		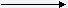		
Coaching engagement		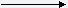		
Coaching acceptability		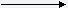		

CGI-I, Clinical Global Impression Scale-Improvement; CGI-S, Clinical Global Impression Scale-Severity of illness; DASS-21, Depression Anxiety Stress Scales – short form; FES, Family Empowerment Scale; FF, Family Focus programme; FQOL, Beach Family Quality of Life Scale; HPAS, Health Promoting Activities Scale.

### Data management

Participant data recorded in REDCap will be managed by Helix (Monash University). Participant intervention engagement and usage data collected via the web application for hosting the FF programme (eg, user profile and preferences, responses entered in module activities, survey reminders and coaching requests) will be stored in a secure cloud database hosted on Amazon Web Services (AWS) infrastructure, ensuring enterprise-grade security, reliability and compliance with regional data residency requirements.

All participant data will be de-identified and assigned a unique code before being used for analysis or reporting. Data sets will be password protected and available only to personnel who have been approved through the ethics application for this study.

### Data analysis

Missing data, participant dropout and loss to follow-up will be minimised through careful trial management. Additionally, the rates of missing data on individual data collection forms will be monitored at each time point throughout the study.

The primary analyses will be undertaken on an intention-to-treat basis. Participant characteristics, participant engagement and intervention usage, and coaching acceptability will be described. A 2 (Group)× 3(Time) repeated measure ANOVA will be applied to compare continuous scores for primary and secondary outcomes between the intervention and comparison groups.

To explore possible attrition biases, we will also conduct analyses to compare retained participants and those lost after baseline assessment, using *t*-tests for continuous measures and chi-square-tests for categorical variables (eg, gender, family socioeconomic and clinical factors). Qualitative data collected using semi-structured interviews (eg, transcripts) will be examined by thematic analysis.

### Risk monitoring

Given the security and privacy procedures outlined in this protocol, there is low–moderate risk to children/families of any harm, inconvenience, or discomfort resulting from participation in the trial. Information regarding potential risks, risk mitigation measures and contact helplines are included in the Participant Information and Consent Form. Further, a trauma-informed approach was applied in designing the online portal where participants access the FF programme. Specifically, features to guide participants on taking breaks from the material and information about supportive helplines and health services were identified to assist participants who might experience feelings of grief or trauma when engaging with the material. If coaches identify any issues of concern regarding a parent, they will recommend they access a relevant community service (eg, present to the local emergency department, contact their treating general practitioner or provide information on helplines). Coaches will have access to discuss any such issues with a senior psychologist on the research team if required. If an assessment shows a score that falls outside the normal limits, participants may be offered feedback, further support and referrals to relevant services.

A data monitoring committee was not considered, as this is a low-risk research study. All standard operating procedures will be documented and provided to research team members who are directly involved in the study. All risks and adverse events, including unexpected side effects, will be monitored and documented by the clinic/trial coordinator, who will act in accordance with the risk management plan. Any significant safety issue or protocol deviation that adversely affects participant safety or the ethical acceptability of the trial will be reported to the investigators and the relevant Human Research Ethics Committee. In the event of new risks or adverse events being identified, the risk management plan will be appended with a course of action. Protocol amendments are communicated to clinical and research team members by the clinic/trial coordinator through email notifications describing the changes and any impacts to operations. These email notifications will include the updated protocol and relevant updated study documents.

## Discussion

This study is the first to evaluate the effectiveness of an online programme with and without 1:1 health professional coaching, designed for families of children who have experienced developmental regression. The trial has several strengths including the pragmatic RCT design in which the trial is embedded within a routine clinical care environment. The blinding of healthcare providers collecting clinical data and outcome data helps to safeguard against potential bias. In addition, online coaching and the use of a flexible online platform to access the FF programme are expected to enhance accessibility. By integrating the insights from families with lived experience, advocates from lived experience agencies, and clinicians into the design of the programme and coaching approach, we have tried to ensure that the interventions are relevant to their needs and that potential risks or concerns are addressed in the design phase. Notwithstanding, given the clinical heterogeneity of the study’s population, it may be necessary to broaden the diversity of health service users (carers) who contributed their experiences and perspectives to the codesign of the online intervention. The additional features (eg, podcasts, audio files and videos) included in the FF programme may encourage engagement which will be measurable via the online portal. Together, these approaches will enable comparisons of the uptake and effectiveness of a self-guided programme for parents of children with developmental regression and a version of the programme that includes individualised coaching.

With this study being the first intervention trial for parents of children with developmental regression, we acknowledge limitations in relation to the study design. These include the lack of participant blinding to their treatment group, and the length and frequency of the follow-up assessments for capturing outcomes. Since baseline assessment may be conducted well before a participant begins accessing the programme or coaching, there is also a risk that variations in the time interval between the baseline assessment and the start of the intervention could allow external factors unrelated to the intervention to influence participants’ responses to intervention. Moreover, participants enrol in the trial one at a time, making it impractical to perform stratified randomisation. Nonetheless, we will conduct subgroup analyses where appropriate to explore potential variation in outcomes.

Participation in the FF programme and coaching requires an internet connection. While the online nature was intended to optimise accessibility, families without a stable internet, adequate data and/or a suitable electronic device may not be able to participate. Despite the implementation of participant engagement and retention strategies, there remains a risk of insufficient sample size. This could result in limited statistical power, potentially constraining the scope and robustness of the planned analyses.

The findings from this trial have the potential to inform future development of interventions for families of children experiencing developmental regression to improve outcomes for parents and their children in terms of mental health, family empowerment, quality of life and participation within their communities. In comparing self-guided FF to FF with coaching, the study will provide preliminary data on the uptake and effectiveness of the different versions of the FF programme for families caring for children with developmental regression, while addressing important questions about the differences between guided and unguided interventions. Additionally, this trial may help shape the design of future interventions for children with developmental regression.

## Ethics and dissemination

The protocol was approved by the Monash Health Research and Ethics Committee (HREC/107806). All participants will access care at the Monash Developmental Regression Clinic for their conditions regardless of study enrolment. Participants will be assigned a study identification number. Data collected from the trial will be systematically stored and managed in a skill loss registry (HREC/91615) if participants have consented to this as well. Study findings will also be disseminated as widely as possible through peer-reviewed publications, conference presentations and lived experience agencies like the Childhood Dementia Initiative and other stakeholders.

### Data availability

The final data set for the study will be available to other investigators on request and approval from Professor Katrina Williams. Interested investigators may be required to provide evidence of Human Research Ethics Committee approval and/or complete a data sharing agreement. To protect participant confidentiality, any data shared with investigators will have identifying information removed.

## Supplementary material

10.1136/bmjopen-2025-105615 online supplemental file 1
