# Supplementary material for: Study protocol for a pragmatic parallel-group randomised controlled trial to evaluate the effectiveness of coaching with an online intervention, compared with the online intervention alone, for families of children who have experienced developmental regression
Source: BMJ Open. 2026 Jun 10;16(6):e105615. doi: 10.1136/bmjopen-2025-105615 (PMC13264943; doi:10.1136/bmjopen-2025-105615)
Supplement: online supplemental file 1 [file bmjopen-16-6-s001.pdf]

A partnership between:

MONASH University   Monash Children's Hospital

Parent/Guardian Consent Form
(to submit via REDCap or as paper copy)

**Project Name:**	Evaluating an early, novel online intervention for primary carers of children with developmental regression: A pragmatic randomised controlled trial
**Project Number:**	107806
**Version Number:**	1.2

Version Date: 7.11.2025

Please fill in the information below and let us know if you would like your family to take part in the study.

**Name of Parent/Guardian**	
**Parent's email address**<br><br>**(please provide the email you will use to log in to the portal and access the Family Focus program)**	
**Name of Child**	
**Child's Date of Birth**	

Please select the relevant box below.

☐ I consent to joining the study.

☐ I do not consent to joining the study.

Optional consents:

i. **Do you give us permission to contact you about other future projects that may be suitable for your family?**

☐ Yes, but we don't have to participate in any of these projects if we don't want to.

☐ No

<Only proceed/display below if option 'I consent to joining the study' is selected>

ii. Do you give us permission to record interactions with you and/or your child? We are interested in reviewing clinical assessments and coaching sessions with your family for research and clinical education purposes.

☐ Yes, video with audio recording, but we can say no later if we no longer want to.

☐ Yes, audio recording only, but we can say no later if we no longer want to.

☐ No, not at all

ii. If your child is a participant of the Victorian Skill Loss Registry, we would like to link data collected from you and your child in this study to data stored in the registry.

☐ I agree

☐ I disagree

☐ Not applicable, my child is not the registry's participant

iii. If you are a participant of Generation V (GenV), we would like to share data collected from you and your child in this study with GenV.

☐ I agree

☐ I disagree

☐ Not applicable, my child is not a GenV participant

By signing on the line (or clicking the *Submit button on REDCap) below, I confirm that:

- I have read the Participant Information, or someone has read it to me in a language I understand
- I understand the contents in information sheet, the purpose and extent of my family's involvement in this project.
- I understand and agree that information about me and/or my family may be discussed during supervision, peer consultation and multidisciplinary meetings among the clinician-researchers involved in the project to support ethical, effective and safe care.
- I have had an opportunity to ask questions and I am satisfied with the answers I have received.
- I understand that transcriptions or de-identification of recordings may be conducted using an approved service provider.
- I understand the risks my family could face because of our involvement in this project.
- I understand that I am free to withdraw at any time during the project without affecting my child's future health or mine
- I voluntarily consent for my family to take part in this research project.
- I understand that the project is required to be carried out in line with the National Statement on Ethical Conduct in Human Research (2025).

- I understand I can download or request a copy of this Information Sheet and Consent Form to keep.

______________________________ ______________________________

Parent/Guardian's signature **Date** ***<if completing on paper>***

____

____

Relationship to Child participant

* If parent/guardian opted to complete the consent form online, the REDCap eConsent Framework will be used to obtain consent and store consent documentation.

Declaration by Witness/Interpreter (if applicable)

I have explained the project to the parent/guardian who has signed above, and believe that they understand the purpose, extent and possible risks of their family's involvement in this project.

**Name of Witness/Interpreter**	

______________________________ ____________________

Witness/Interpreter's signature **Date**

Declaration by researcher:

I have explained the project to the parent/guardian who has signed above, and believe that they understand the purpose, extent and possible risks of their family's involvement in this project.

**Name of Researcher**	

______________________________ ____________________

Researcher's signature **Date**

Note: *All parties signing the Consent Form must date their own signature*
