# Supplementary material for: Study protocol for a pragmatic parallel-group randomised controlled trial to evaluate the effectiveness of coaching with an online intervention, compared with the online intervention alone, for families of children who have experienced developmental regression
Source: BMJ Open. 2026 Jun 10;16(6):e105615. doi: 10.1136/bmjopen-2025-105615 (PMC13264943; doi:10.1136/bmjopen-2025-105615)
Supplement: Supplementary file 2 [file bmjopen-2025-105615.reviewer_comments.pdf]

# PEER REVIEW HISTORY

BMJ Open publishes all reviews undertaken for accepted manuscripts. Reviewers are asked to complete a checklist review form and are provided with free text boxes to elaborate on their assessment. These free text comments are reproduced below.

## ARTICLE DETAILS

### Title (Provisional)

Study protocol for a pragmatic parallel-group randomised controlled trial to evaluate the effectiveness of coaching with an online intervention, compared to the online intervention alone, for families of children who have experienced developmental regression

### Authors

Sim, Wan Hua; Leo, Monica; Bourke-Taylor, Helen M; Bernie, Charmaine; Brignell, Amanda; Ure, Alexandra; Furley, Kirsten; Fahey, Adele Maree; Bhopti, Anoo; Odgers, Sorcha; Picking, Leah; Xie, Jue; Wu, Ling; Yap, Marie B. H.; Williams, Katrina

---

## VERSION 1 - REVIEW

---

| | |
|---|---|
| **Reviewer** | **1** |
| **Name** | **Turnbull, Deborah** |
| **Affiliation** | **University of Adelaide, Psychology** |
| **Date** | **03-Mar-2026** |
| **COI** | **None** |

---

The report is very well presented and comprehensive. It covers all the relevant aspects of the trial and the authors are transparent about the limitations. The intervention has been designed with consumer group involvement. My suggestion is that there should be a stronger rationale for linking the program with the main outcome of parental stress. What is the intervention logic or theory of change? I think that the reader would like to know more about the clinic and the referral process. This is a publicly funded clinic? Who refers to the clinic? Finally, the eligibility criteria re suspected or confirmed developmental regression is vague. I think that the explanation can be expanded. Is this a clinical diagnosis? What is the diagnosis code being applied?

---

## VERSION 1 - AUTHOR RESPONSE

Reviewer’s Comments to the Author:

***I think that the reader would like to know more about the clinic and the referral process. This is a publicly funded clinic? Who refers to the clinic? Finally, the eligibility criteria re suspected or confirmed developmental regression is vague. I think that the explanation can be expanded. Is this a clinical diagnosis? What is the diagnosis code being applied?***

**Response:** We thank the reviewer for highlighting areas where additional detail would assist the reader's understanding about the clinic and referral process. In response, we have revised the Methods section to more clearly describe the clinic, referral pathways, and eligibility criteria.

We now state that the Developmental Regression clinic is a publicly funded tertiary service supported by a state public hospital and a Medical Research Future Fund (MRFF) grant from the Australian Government, and clarify that referrals are accepted from GPs or other medical specialists across Victoria (pages 7-8).

We have also expanded on and operationalised the definition of suspected developmental regression as a clinical presentation (rather than a single coded diagnosis) (page 8), and added detail about the multidisciplinary assessment process and potential explanatory diagnoses considered.

We believe these revisions address the reviewer's concerns and provide readers with a clearer understanding of how children are referred to, and assessed within, the clinic. The relevant additions and revisions are presented below.

> **(Pages 7-8) Study setting**
> The clinic is a public specialist tertiary service jointly funded by the hospital and a Medical Research Future Fund (MRFF) grant from the Australian Government. Referrals are accepted from GPs or other medical specialists in Victoria. Eligibility for the clinic is based on suspected developmental regression, defined as parent- or clinician-reported loss of previously acquired skills in at least one developmental domain (communication, motor, social engagement, or daily living) within the preceding 18 months. This is a clinical presentation rather than a discrete entity with a single diagnostic code, and it serves as the trigger for a structured multidisciplinary assessment to clarify the underlying aetiology. Regression is operationalised through detailed developmental history, supported where possible by documented prior functioning (e.g. reports, home videos, or clinician observations).
>
> Children referred to the clinic are seen by a multidisciplinary team consisting of a paediatrician, an occupational therapist clinic coordinator, a neuropsychologist and/or a speech pathologist. Each child undergoes a comprehensive assessment consisting of

parent- report, direct observation and standardised measures, with the specific assessment battery tailored to each child's age, abilities and presenting features. The multidisciplinary team conducts weekly case conferences with neurology and genetic specialists to plan first-line investigations and early management. Children are reviewed at approximately 8 weeks, 6 months and 12 months. At each review, investigation results and clinical progression are reviewed, and further targeted investigations are recommended where an explanatory diagnosis remains unclear. Possible explanatory diagnoses considered include autism, neurodegenerative conditions (e.g. Batten disease), developmental epileptic encephalopathies (DEE), recognised genetic conditions (e.g. Rett syndrome), and other rare or emerging genomic or mitochondrial conditions, including possible phenotypic expansions). Where autistic features emerge over the follow-up period, an autism-focused assessment may be undertaken alongside standard developmental assessments and medical investigations. The clinic explicitly seeks to minimise diagnostic overshadowing by continuing to investigate other potential causes of regression even when autistic features are present.

**(Pages 8-9) Eligibility criteria**

Participants are not eligible if: (i) the child's history of loss of skills cannot be corroborated through developmental surveillance information, clinical assessment, or review of video/audio recordings, or (ii) the regression is judged to have occurred primarily in the context of significant environmental adversity such as documented abuse, neglect or severe household adversity, or (iii) the child's condition warrants prioritisation of medical intervention and stabilisation over research participation.

Finally, in addition to addressing the reviewer's comments, we have updated several details about the trial to reflect changes since the manuscript's original submission in May 2025. Specifically, we have:

i) Updated the trial recruitment start and end dates (Abstract and page 9)
ii) Clarified the schedule for the collection of data that do not comprise trial outcome measures (e.g. skill regression information, developmental history, physical and medical examination), which will now be collected and analysed only at baseline (Table 2, pages 19-20)

iii) Corrected an error in Table 2 where “CGI-I” was listed twice; once instance has been amended to “CGI-S” to indicate the Clinical Global Impression Scale-Severity of illness (Table 2, pages 19-20).
